# LIO-SAM++: A Lidar-Inertial Semantic SLAM with Association Optimization and Keyframe Selection

**DOI:** 10.3390/s24237546

**Published:** 2024-11-26

**Authors:** Bingke Shen, Wenming Xie, Xiaodong Peng, Xiaoning Qiao, Zhiyuan Guo

**Affiliations:** 1 National Space Science Center, Chinese Academy of Sciences, Beijing 100190, China; shenbingke22@mails.ucas.ac.cn (B.S.); pxd@nssc.ac.cn (X.P.); qiaoxiaoning19@mails.ucas.ac.cn (X.Q.); guozhiyuan19@mails.ucas.ac.cn (Z.G.); 2 University of Chinese Academy of Sciences, Beijing 100049, China; 3 Hangzhou Insititute for Adcanced Study, Chinese Academy of Sciences, Hangzhou 310024, China

**Keywords:** lidar-inertial SLAM, semantic information, association optimization, keyframe selection

## Abstract

Current lidar-inertial SLAM algorithms mainly rely on the geometric features of the lidar for point cloud alignment. The issue of incorrect feature association arises because the matching process is susceptible to influences such as dynamic objects, occlusion, and environmental changes. To address this issue, we present a lidar-inertial SLAM system based on the LIO-SAM framework, combining semantic and geometric constraints for association optimization and keyframe selection. Specifically, we mitigate the impact of erroneous matching points on pose estimation by comparing the consistency of normal vectors in the surrounding region. Additionally, we incorporate semantic information to establish semantic constraints, further enhancing matching accuracy. Furthermore, we propose an adaptive selection strategy based on semantic differences between frames to improve the reliability of keyframe generation. Experimental results on the KITTI dataset indicate that, compared to other systems, the accuracy of the pose estimation has significantly improved.

## 1. Introduction

SLAM (Simultaneous localization and mapping) technology involves a robot constructing environment maps and estimating its position in an unknown environment by collecting data from its surrounding environment using its mounted sensors. Due to the continuous development of intelligent technology, SLAM technology has gained widespread attention and application in robotics and autonomous driving. SLAM can be categorized into Lidar-based and visual-based approaches, depending on the type of sensors used for environmental perception. Visual SLAM captures abundant visual data through cameras, providing color and texture information of the scene; however, it is susceptible to changes in illumination. Conversely, LIDAR (Light Detection and Ranging) is capable of accurately measuring distance and remains unaffected by varying illumination conditions, rendering it more commonly utilized in large-scale environments. Notably, the laser-based multi-sensor fusion can further enhance environmental perception and serve as a crucial method for improving the performance of SLAM systems.

In recent years, many solutions have been proposed for lidar-inertial SLAM, such as LEGO-LOAM (Lightweight and Ground-Optimized Lidar Odometry and Mapping) [1], LIO-SAM (Lidar Inertial Odometry via Smoothing and Mapping) [2], and FAST-LIO (A fast robust LiDAR-inertial odometry) [3]. These methods perform well in some scenes but may be disturbed in the real word. Most of these works select the associated points in the feature-matching process based only on the nearest-neighbor distance information. However, this way of association does not consider the effects of point positions and densities between point cloud frames, which results in a lack of robustness in the pose transformation. In addition, the point cloud maps of the environment generated by these algorithms only contain geometry information and lack semantic descriptions of the objects in the scene, making it difficult to understand the scene in depth. As neural networks advance in the field of point cloud semantic segmentation, researchers have integrated semantic information into LIDAR-based SLAM frameworks. Semantic-aided LiDAR SLAM, such as SUMA++ (Efficient LiDAR-based Semantic SLAM) [4] and SA-LOAM (Semantic-aided LiDAR SLAM) [5], use semantic segmentation networks to obtain the semantic labels of the scene, which provides additional constraints for the point cloud alignment process and reduces the dependence on the environment geometry. Although these methods have made some progress, the performance of these can still be deficient when facing environmental changes in the scene.

In this paper, we propose a lidar-inertial SLAM with association optimization and keyframe selection. We optimize the feature association by combining geometric and semantic information and reduce trajectory drift by adaptively generating keyframes according to scene changes. The main contributions presented in this work can be summarized as follows:

(1) We propose a feature association method based on neighborhood normal vector consistency, which uses local region geometric information to filter the nearest neighbor feature points to reduce the problem of inaccurate feature point association in point cloud registration.
(2) We propose a semantic-assisted point cloud registration method for pose estimation, which constructs a weighted cost function based on semantic attributes to achieve more reliable odometry data association and improve pose estimation accuracy.
(3) We propose a semantic-based keyframe optimization method to adaptively generate keyframes through the difference of semantic information between frames, which reduces the probability of losing the valid point cloud frame.

## 2. Related Work

### 2.1. Feature Association

In SLAM systems, the process of scan registration is obtaining the pose transformation by aligning two successive point cloud frames or the current point cloud frame with the established map. Geometry-based association methods often employ geometry primitives, such as points, lines, planes, or curvature features, to facilitate rapid matching. In LOAM [6], point cloud curvature is computed to categorize points as either surface or corner features. Residual equations are then formulated from the distances of corner points to lines and surface points to planes, which are utilized to optimize pose estimation via the Levenberg-Marquardt method. Many subsequent algorithms continue to adopt this feature-matching approach, such as LeGO-LOAM [1], which reduces the complexity of computation by segmenting the ground points of the point cloud frames and then clustering them to obtain labels, and using the label information as the constraints for inter-frame matching. LIO-SAM [2] extended LeGO-LOAM by removing the frame-to-frame matching part and incorporating IMU pre-integration and GPS measurements factor within the factor graph optimization. FAST-LIO [3] also extracts the corner and surface point features and proposes a new Kalman gain formula to achieve efficient computation. In an effort to further improve the reliability of solving pose transformation, some works began to focus on the categorization of features. Guo et al. [7] introduced an approach using Principal Components Analysis (PCA) to distinguish between corner and surface points utilizing the properties of the points. Similarly, MULLS [8] extracted various features based on PCA, which proposed a multi-metric linear least squares method for iterative optimization of pose estimation. Meng et al. [9] proposed an adaptive feature extraction method for different scenes, which adjusts planar and linear feature thresholds according to local environmental properties and constructs multiclass cost functions for distinct categories of features.

The above research enhanced the precision of feature point association by categorizing feature points and seeking within the identical category. However, we consider that feature points of the same class are still associated with nearest neighbors, which affects the accuracy of the association in cases when point cloud frames and maps do not overlap completely. Therefore, unlike these feature association optimization methods, our approach improves the reliability of feature association by expanding the scope of the nearest neighbor search and combining it with normal vector consistency for filtering.

### 2.2. Pose Estimation Combining Semantic Constraints

Semantic information plays a crucial role in environment understanding. High dimensional semantic information can provide more a priori conditions for the system, which can construct constraints that help optimize the state estimation and achieve more accurate point cloud frame-to-map matching. SuMa++ [4], based on the SuMa, introduces semantic information to optimize the pose estimation by adjusting the residual weights using semantic consistency. When the semantic labels at the corresponding positions in the frame and the map are the same, the residual weight is increased to improve the registration robustness. Similarly, Zhao et al. [10] and Du et al. [11] proposed further use of geometry features to construct residuals only if the semantic labels are identical.

These studies assign weight coefficients to the associated point pairs based on semantic label consistency, thereby enhancing the precision and stability of SLAM and showing the significant potential of using semantic information as a priori conditions. In contrast to the methods, our approach considers the challenge of inaccurate semantic segmentation in boundary areas and avoids complete dependence on the semantic labels of associated feature points.

### 2.3. Keyframe Selection

SLAM systems are often required to process a vast quantity of lidar data, resulting in significant algorithmic computational load due to the rapid lidar point cloud updates and the information overlap between frames. To solve the problem, only keyframes instead of all frames are usually utilized in the back-end optimization process, thus ensuring accuracy while improving computational efficiency. Therefore, a reasonable keyframe selection criterion is necessary. Currently, the criteria used to select keyframes may be classified into two principal categories: heuristic-based methods and information theoretic methods.

Heuristic-based methods usually determine keyframes by time or pose changes. For example, LIO-SAM [2] and SVO [12] calculate the translation distance and rotation Angle of the robot pose estimation between the current frame and the last keyframe, selecting the current frame as a keyframe upon exceeding a defined threshold of change. ORB-SLAM [13] extracts the keyframes by checking the number of frames between two frames. DSO [14] extracts the new keyframes by combining the field of view, camera position, and exposure time variations as criteria for selecting new keyframes. VINS-MOON [15] employs another heuristic strategy for keyframe selection, which is detecting whether the feature count in the current frame falls within a set threshold range. In contrast, information theoretic methods have more stringent metrics. These methods decide whether to insert a new keyframe by evaluating the uncertainty or quality of the pose estimate of the current frame. Kuo et al. [16] select keyframes based on the differential entropy reflecting the uncertainty of the current camera estimation of the pose concerning the historical keyframe. Jiao et al. [17] select keyframes by computing the information matrix and assessing the convergence of the state estimation problem. Based on this work, InfoLa-SLAM [18] considers the information between neighboring frames and selects keyframes more comprehensively by constructing the Fisher information matrix.

Although the above keyframe selection methods are effective in reducing redundancy, they primarily depend on geometric information or the outcomes of geometric optimization. This approach will overlook the characteristics of scene changes. As a result, it may fail to capture significant details, potentially introducing bias into the estimated trajectories. Considering semantics can describe the scene effectively, we use the semantic information to focus on the scene changes, generating more reasonable keyframes.

## 3. Method

**Figure 1:**
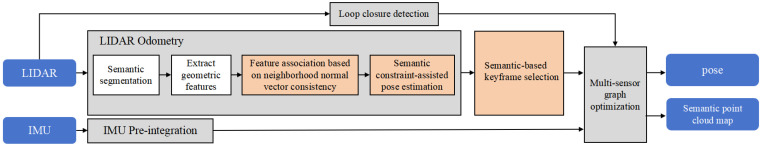
The structure of the LIO-SAM++.

The architecture of the proposed lidar-inertial semantic SLAM LIO-SAM++ is depicted in Figure 1. The input is point cloud data from the LiDAR and high-frequency pose data from the IMU, and the output is the state estimation and an environment map with semantic information. This process has several stages, including IMU pre-integration, lidar odometry, keyframe selection, loop closure detection and graph optimization.

(1) During the IMU pre-integration phase, the IMU measurements between two frames of LiDAR data are pre-integrated;
(2) During the lidar odometry phase, semantic segmentation is applied to the point cloud frames via a pretrained network to get semantic labels; Subsequently, corner points and surface points are extracted from these frames, with the IMU pre-integration providing an initial pose estimate. The pose of the current frame is obtained by minimizing the residuals with semantics and geometry. For details, refer to Section 3.1;
(3) During the keyframe selection phase, keyframes are generated based on the scene semantic changes and estimated pose transformation for back-end graph optimization. For details, refer to Section 3.2;
(4) During the loop closure detection phase, potential loop closure relationships are found by detecting historical keyframes to establish loop closure constraints;
(5) During the graph optimization phase, lidar odometry factors, IMU pre-integration factors and loop closure detection factors are constructed. These factors are used to update the state of system estimation through factor graph optimization and the output of accurate pose estimation at the rate of the IMU. Meanwhile, global consistent point cloud maps are generated by transforming the keyframe collection to the world coordinate system and fusing them with the constructed global map;

In the next sections, we present detailed information about each module.

### 3.1. Lidar Odometry

The lidar odometry achieves the registration of a point cloud by fusing the lidar and IMU data and then estimates the relative pose. Given a LiDAR point cloud $p^{t}$ at time stamps *t* and the local map point cloud $M^{t}$ the transformation matrix *T* between the point cloud frame and the local map is estimated by feature matching.

(1) $$T=\underset{T}{\arg\min}\sum_{i}{∥m_{j}^{t}-Tp_{i}^{t}∥}^{2}$$

where $m_{j}^{t}$ and $p_{i}^{t}$ is the corresponding points between the local map and point cloud frame.

#### 3.1.1. Feature Extraction

We incorporate a semantic segmentation network for point clouds into the SLAM framework to obtain semantic information. The goal of semantic segmentation of point clouds is to categorize individual points, thereby getting their semantic labels. Considering adapting to the frame rate requirements of sensors and meeting real-time requirements, in this paper, we use RangeNet++ [19] to segment the de-distorted point cloud frames to obtain semantic information in the environment. RangeNet++ was trained on the SemanticKITTI dataset [20] and is capable of distinguishing between 22 different semantic categories, including roads, vegetation and buildings, among other objects.

We extract features from the segmented current lidar point cloud frames to describe their geometric attributes, distinguishing corner and surface feature points based on curvature calculations [2]. To avoid the clustering of feature points, the lidar scan line is segmented into several parts, ensuring that points within proximity on the same scan line are not processed repeatedly. The curvature *c* is obtained by calculating the spatial variation of the surrounding points:

(2) $$c=\frac{1}{|S|\cdot ∥r_{i}∥}{∥\sum_{j\in S,j\neq i}\left(r_{i}-r_{j}\right)∥}^{2}$$

where *S* represents the collection of points located on either side of the current point along the same lidar scan line, $r_{i}$ and $r_{j}$ denote the vectors from the origin of the coordinate system to the points $p_{i}$ and $p_{j}$. A point is designated as a corner feature if its curvature exceeds a predefined threshold, whereas a point below this threshold is classified as a surface feature.

Ultimately, the extracted corner features $F_{i}^{e}$ and surface features $F_{i}^{s}$ from the point cloud of each frame, which are combined into a feature set $F_{i}=\left\{F_{i}^{e},F_{i}^{s}\right\}$. This set represents the current frame and is utilized for subsequent registration of point clouds.

#### 3.1.2. Feature Association

The accuracy of pose estimation of SLAM depends on the accuracy of point cloud matching, so correct feature association is crucial. Traditional feature association methods usually directly obtain the nearest neighbors of corner and surface feature points and use these points to construct linear and planar constraints for nonlinear optimization. However, this assumption that the nearest neighbors are the associated feature points is not completely reliable because it does not guarantee that the nearest neighbors are the correctly associated points. The reason for this problem is that the search for nearest neighbors as associated points requires that the corresponding points nearly overlap with each other. In contrast, the transformed point cloud and the local maps cannot satisfy the nearly overlapping condition due to the initial value impact and the difference in point density. Erroneous point associations can adversely impact the precision of matching results, while conventional methods often overlook the geometry structural information in point clouds. However, this geometric information is crucial to find the correct association between points. Therefore, we propose to use geometric information for feature point selection by modeling the geometric distribution around each feature point and introducing a feature association approach based on local normal vector description to find more accurate associated features.

Firstly, the feature point set $F_{i}$ from the current point cloud scan is mapped into the world coordinate system using the pose transformation $T_{i}^{imu}$ obtained from IMU pre-integration. In the transformed coordinate system, for each feature point $f_{i}$, 15 candidate matches $MC_{i}$ are found through nearest neighbor search in the feature point set $M_{i}$ of the local map. Next, the set of nearest points around the feature point $f_{i}$ is searched in the point cloud scan to form a neighborhood R to quantify the geometric similarity between the points. Finally, obtain the set of 5 nearest neighbor points $MD_{i}$ by filtering based on the geometric similarity and used to construct the residual optimization to ensure the point cloud matching accuracy better.

To quantify the geometric similarity between points, we propose a method for describing local areas that utilize a normal vector. The method calculates normal vectors through the covariance matrix and thus combines the nearest distance information to achieve the association of feature points. For each feature point $f_{i}$ in a point cloud, we first determine the average position $\bar{f}$ of the points within the neighborhood *R*. Subsequently, leveraging the positional variance of these neighboring points from the mean, we calculate the covariance matrix *C*. Finally, we perform an eigendecomposition of *C* to extract its eigenvalues λ and corresponding eigenvectors. The eigenvector corresponding to the smallest eigenvalue is the normal vector of the local region. The unit normal vector $\hat{n}$ is defined by the formula:

(3) $$\bar{f}=\frac{1}{N}\sum_{i=1}^{N}f_{i}$$

(4) $$C=\frac{1}{N}\sum_{i=1}^{N}\left(f_{i}-\bar{f}\right){\left(f_{i}-\bar{f}\right)}^{T}$$

(5) $$\mathrm{Cn}=\lambda_{min}n$$

(6) $$\hat{n}=\frac{n}{∥n∥}$$

where $f_{i}$ represents the coordinates of the ith point in the neighborhood, *N* is the total count of points in the neighborhood, and $\lambda_{min}$ refers to the eigenvalue of the smallest eigenvalue, *n* denotes the normal vector.

According to Equation (6), we compute the local unit normal vector ${\hat{n}}_{1}$ for the points in the point cloud scan and the local unit normal vector ${\hat{n}}_{2}$ for each candidate matching point in the map. Subsequently, we compare the difference between ${\hat{n}}_{1}$ and ${\hat{n}}_{2}$. If the local normal vector direction of a point in a point cloud scan is consistent with that of a matching point in the local map, it indicates that the two points may lie on the same plane, and the match is retained. The consistency of normal vectors is defined by the angular difference between the vectors:

(7) $$\theta =\arccos({\hat{n}}_{1}\cdot {\hat{n}}_{2})$$

where ${\hat{n}}_{1}$ and ${\hat{n}}_{2}$ are the unit normal vectors corresponding to the point cloud scan and the local map, respectively. Consistency of the normal vector is indicated if the angle θ is below a predetermined threshold.By calculating the local unit normal vector consistency between the point cloud frames and the corresponding points of the local map, five more accurate matching points are selected from the 15 candidate matching points. These five matching points will be used to construct geometric residual and semantic attribute constraints to improve the reliability of the matching results.

The feature association strategy based on normal vector consistency has higher robustness than the traditional method that only relies on nearest neighbor search. The traditional methods may produce false matches due to noise. In contrast, the proposed method can effectively filter out incorrect matches and preserve points with similar geometric features, thus improving the accuracy of the matches.

The process of the association point selecting method is shown in the Algorithm 1.

#### 3.1.3. Pose Estimation

In SLAM algorithms, pose estimation is performed by optimizing the constraint residuals of the associated feature points, and the calculation results are directly related to the accuracy of the final pose estimation. Traditional constraint construction methods often rely on the established feature association relationships on which constraint residuals are computed separately for the associated corner and surface feature points to minimize the geometric error between the point cloud and the map. However, the association relationships of feature points in these methods are susceptible to occlusion or dynamic objects, which reduces the accuracy of the associations. To reduce the impact of mismatching on the optimization process, this paper introduces semantic information to assist map matching during the geometric constraint construction process, thereby improving the robustness of the optimization process. The method uses semantic information to provide an additional basis for the judgment of matching pairs, which can reduce the impact of semantic inconsistency points on the optimization.
**Algorithm 1** Feature association based on neighborhood normal vector consistency**input:** current point cloud frame set $L=\{p_{1}^{l},p_{2}^{l},\ldots ,p_{m}^{l}\}$, local map point cloud set $M=\{p_{1}^{m},p_{2}^{m},\ldots ,p_{n}^{m}\}$, the pose transformation *T* obtained from IMU pre-integration.
**output:** The set $P_{s}$ of 5 points that satisfy the requirement
1. for $p_{i}^{l}$ in *L* do
2.    lidar2map($p_{i}^{l}$,$p_{i}^{l\to m}$)
3.    $M_{15}$← kdMapNearest($p_{i}^{l\to m}$,15)
4.    for $p_{i}^{m}$ in $M_{15}$ do
5.       $M_{10}$← kdMapNearest($p_{i}^{m}$,10)
6.       $L_{10}$← kdLidarNearest($p_{i}^{l\to m}$,10)
7.       if(checkNormalConl($L_{10}$,$M_{10}$,*T*))
8.           add $p_{i}^{m}$ to $P_{s}$
9.           if($P_{s}$.size==5)
10.              break;
11.          end if
12.      end if
13.   end for
14. end for

The method flow is as follows: firstly, the distance residuals are constructed separately for corner and surface feature points. Next, the semantic consistency probability is calculated and the residuals are weighted using this probability. Finally, the weighted residuals are optimized as the cost function of the lidar odometry.

For corner points, the residual is defined as the distance from the feature point of the current frame to the line formed by the 2 nearest neighbors of the corresponding 5 points in the local map. For surface points, the residual is defined as calculating the distance from the feature point of the current frame to the plane formed by the 3 non-collinear points among the corresponding 5 points in the local map. The corner point residuals are calculated as follows:

(8) $$d_{e}=\frac{|\left(f_{i,k}^{e}-f_{u,m}^{e}\right)\times \left(f_{i,k}^{e}-f_{v,m}^{e}\right)|}{|f_{u,m}^{e}-f_{v,m}^{e}|}$$

where $d_{e}$ denotes the distance from the corner feature point to the line, $f_{i,k}^{e}$ is the corner point in the current point cloud frame, and $f_{u,k}^{e}$ and $f_{v,k}^{e}$ are the two nearest neighbors corner points in the local map.

The surface point residuals are calculated as follows:

(9) $$d_{s}=\frac{|\left(f_{i,k}^{s}-f_{u,m}^{s}\right)\cdot \left(\left(f_{u,m}^{s}-f_{v,m}^{s}\right)\times \left(f_{u,m}^{s}-f_{w,m}^{s}\right)\right)|}{|\left(f_{u,m}^{s}-f_{v,m}^{s}\right)\times \left(f_{u,m}^{s}-f_{w,m}^{s}\right)|}$$

where $d_{s}$ denotes the distance from the surface point to the plane, $f_{i,k}^{s}$ is the surface point in the current point cloud frame, and $f_{u,m}^{s}$, $f_{v,m}^{s}s$, and $f_{w,m}^{s}$ are the three nearest-neighbour noncollinear points in the local map.

For semantics, the traditional approach is to reduce the error caused by mismatching by directly identifying the points that are inconsistent with the semantic attributes of the current point. However, in this paper, considering the possible inaccuracy of semantic segmentation, especially at the edges, instead of adopting the constraint construction by considering only the points with the same semantic categories [5], the residuals are weighted by calculating the semantic weights. Specifically, semantic weights are first obtained by calculating the probability of semantic consistency between the current point and its five matches selected by the normal vector consistency-based feature association method. Then, this semantic weight is utilized to weight the residuals to mitigate the effect of outliers. The semantic label $l_{i}^{c}$ of the current point $f_{i}$ has the semantic distribution $MS_{i}={\left\{l_{k}^{m}\right\}}_{k=1}^{N}$ corresponding to the set of matching points $MD_{i}$, and the semantic consistency judgment is expressed as follows.

(10) $$n_{k}^{i}=\left\{\begin{array}{c} 1\;\mathrm{if}\;\;l_{i}^{c}=l_{k}^{m}\\ 0\;\mathrm{otherwise} \end{array}\right.$$

The weighted corner point residuals $d_{e}^{\prime }$ and surface point residuals $d_{s}^{\prime }$ are formulated as follows:

(11) $$d_{e}^{\prime }=d_{e}\times \frac{\left(\sum_{k=1}^{N}n_{k}^{i}\right)}{N}$$

(12) $$d_{s}^{\prime }=d_{s}\times \frac{\left(\sum_{k=1}^{N}n_{k}^{i}\right)}{N}$$

where $d_{e}$ denotes the distance residuals of corner points, $d_{s}$ denotes the distance residuals of surface points, *n* denotes the number of points in the matched points that are consistent with the semantic attributes of the current point, and *N* denotes the number of matched points.

The introduction of semantic weights changes the objective of residual computation, enabling the optimization to not only depend on geometry distances but also consider the consistency of semantic attributes. This method effectively reduces the adverse effect of geometric similar but semantic inconsistent points on the optimization and then improves the accuracy of the solution. When semantic inconsistencies occur, the contribution of wrong matches to the optimization objective function can be reduced by multiplying the weights by less than one.

Ultimately, the optimal transformation matrix is solved by constructing a cost function with the weighted residuals of corner and surface point distances and then optimizing this cost function through the Gauss-Newton optimization method. The optimization formula is as follows:

(13) $$T^{*}=\underset{T^{*}}{\arg\min}\left\{\sum_{f_{i,k}^{e}\in F_{i}^{e}}d_{e}^{\prime }+\sum_{f_{i,k}^{s}\in F_{i}^{s}}d_{s}^{\prime }\right\}$$

where $T^{*}$ denotes the optimal transform obtained by solving.

### 3.2. Semantic-Based Keyframe Selection

During SLAM, there is a wide overlap in consecutive frames, and optimizing all the frames would lead to data redundancy and a huge computational burden. Hence, keyframe selection is required. Heuristic methods usually select keyframes by calculating the relative motion position. Specifically, when the pose transformation between the current frame and the previous keyframe exceeds a certain translation or rotation threshold, the current frame is set as a new keyframe, while other frames between the two keyframes are discarded. This process can be described as follows: for the previous keyframe and the new point cloud frames $P_{1}$ and $P_{2}$, the pose transformations obtained through the odometry are $T_{1}$ and $T_{2}$, and the new point cloud frames are defined as keyframes when the difference between $T_{1}$ and $T_{2}$ satisfies the condition $∥t_{12}∥>\tau_{t}$ or $∥\theta_{12}∥>\tau_{r}$. Where $\tau_{t}$ and $\tau_{r}$ represent the translation and rotation thresholds, $∥t_{12}∥$ and $∥\theta_{12}∥$ represent the translation vector norm and the rotation angle norm. We ensure by using distance and angle thresholds that the system selects a new keyframe only when the pose has sufficient translation or rotation.

However, the selection of keyframes based only on pose changes may ignore frames with drastic changes in the environment structure, resulting in the loss of keyframes. This phenomenon occurs because drastic changes in the scene are not immediately reflected as significant changes in the pose, resulting in the keyframe selection process not capturing these changes in time and thus affecting the optimization results. When the scene changes rapidly, more keyframes can help the SLAM system to adapt to these changes better and avoid error accumulation caused by drastic changes in the scene.

In addition, in complex environments, the presence of dynamic objects increases the uncertainty and noise in the SLAM system, causing discontinuity in the observation data and affecting the accuracy of the pose estimation. Therefore, in the scenes with dynamic objects, selecting more stable keyframes can make the pose estimation process smoother, reduce the error association, and reduce the phenomenon of pose jumps due to dynamic objects.

Therefore, given the limitations of heuristics at the level of understanding the environment, we propose to introduce a semantic description of the scene. Usually, the distribution of objects in the same scene region is relatively stable, and the semantic class of objects can be used to characterize the region. Since the semantic changes between neighboring point cloud frames can reflect the apparent differences in the environment, it enables the SLAM system to capture the environmental changes more accurately in complex dynamic environments and rapidly changing scenes. Therefore, we introduce semantic label information in the keyframe selection stage to optimize the keyframe selection strategy through semantic change detection and semantic stability detection. Compared with heuristics, our approach better reflects the scene characteristics.

Before describing our keyframe selection method in detail, the relevant statistical background is first provided. The KL (Kullback-Leibler) divergence is derived from the concept of information entropy, which is used to describe the amount of information that is available when generating a random variable from a distribution. For a random variable *x* given a probability distribution P(x), the information entropy H(P) is defined as follows:

(14) $$H\left(P\right)=-\sum_{x}P\left(x\right)\log P\left(x\right)$$

Based on information entropy, KL divergence is used as a measure of the relative difference between two probability distributions. Assume that for a random variable *x*, there exist two probability distributions P(x) and Q(x), and assume that P(x)>0 and Q(x)>0 hold for all *x*. If *x* is a discrete random variable, define the KL dispersion from *P* to *Q* to be:

(15) $$D_{KL}\left(P∥Q\right)=\sum_{x}P\left(x\right)\log\frac{P(x)}{Q(x)}$$

In our keyframe selection strategy, the points numbers of different semantic categories in the two lidar frames are modeled as a discrete random variable, requiring the probability distribution of each semantic category to be non-negative and normalized, i.e., the probability of all categories sums up to 1, and ensuring that the probability value of each semantic category is greater than 0 in the previous frame to ensure that the calculation of the KL divergence is valid. First, the semantic distribution of the current frame is established by calculating the number of points of each semantic category. We then use KL divergence to describe the difference in semantic probability distributions between the current frame and the previous frame.

The semantic distribution is defined as the percentage of the number of different semantic points and is calculated as follows:

(16) $$P_{i}=\frac{n_{i}}{N}$$

where *N* is the total count of points in the point cloud and $n_{i}$ signifies the count of points with semantic label *i* within the point cloud.The KL divergence is calculated as follows:

(17) $$D_{KL}=\sum_{i=1}^{n}P_{i}^{c}\log\left(\frac{P_{i}^{c}}{P_{i}^{l}}\right)$$

where $P_{i}^{c}$ and $P_{i}^{l}$ are the frequency of each semantic class object in the current and previous frames. A larger KL divergence indicates a larger semantic difference between the current frame and the previous frame, and then the current frame is more likely to be selected as a keyframe. Compared with keyframe selection relying only on pose changes, modeling semantic information using KL divergence can more acutely capture changes in object categories and distributions in the scene, especially when the scene changes drastically, but the pose changes are not obvious.

Secondly, we exploit semantic information to select point cloud frames that contain a larger number of absolute static object labels. The semantic stability score SS of the current frame is indicated by calculating the proportion of static objects relative to dynamic objects with the following formula:

(18) $$SS=\frac{n_{s}}{n_{d}+1}$$

where $n_{s}$ is the number of absolute static objects, and $n_{d}$ is the number of dynamic objects. The semantic stability score reflects the static degree of the current frame. It effectively reduces the interference caused by dynamic objects by favoring the selection of frames with higher static as keyframes.

Next, based on the calculated semantic distribution probability and stability score, different semantic weights $W_{se}$ are defined to adjust the threshold of keyframe selection based on translation and rotation in the traditional geometry method. The equations for calculating the translation and rotation thresholds are as follows:

(19) $$m=\left\{\begin{array}{c} 0\;\mathrm{if}\;\;SS>1*t_{ss}\\ 1\;\mathrm{if}\;\;1*t_{ss}<SS<1.2*t_{ss}\\ 2\;\mathrm{if}\;\;SS>1.2*t_{ss} \end{array}\right.$$

(20) $$W_{se}=0.9^{m}\times 1-D_{KL}$$

(21) $$\tau_{t}^{\prime }=W_{se}\times \tau_{t}$$

(22) $$\tau_{r}^{\prime }=W_{se}\times \tau_{r}$$

where $\tau_{t}$ and $\tau_{t}^{\prime }$ are the original and adjusted translation thresholds; $\tau_{r}$ and $\tau_{r}^{\prime }$ are the original and adjusted rotation thresholds, and the parameter m is related to the semantic stability score SS. To dynamically change the selection of keyframes according to the static degree of the scene, we set the threshold tss to 10. In addition, to balance the system accuracy and computational efficiency and to ensure sufficient spatial and perspective changes between keyframes, the translation distance threshold $\tau_{t}$ is set to 1.0 meters, and the rotation angle threshold $\tau_{r}$ is set to 0.2 radians. By introducing semantic weights, keyframes can be selected more flexibly than traditional geometric thresholds.

The use of semantic information to select keyframes provides a more accurate understanding of environmental changes. It improves the ability of the system to perform in different environments compared to selecting keyframes using only pose transformations.

Finally, by judging the relationship between the interframe pose change and the adjusted threshold, it is decided whether the current frame is a keyframe or not: if the change is larger than the threshold, then the frame is selected as a keyframe. Otherwise, the current frame is discarded. The selected keyframes are not only used for factor map optimization but are also accumulated to build local and global maps gradually. The more comprehensive keyframe selection method can effectively improve the accuracy and robustness of the position estimation.

The keyframe selection method process is shown in Algorithm 2.
**Algorithm 2** Semantic constraint-assisted pose estimation**input:** current frame point cloud set $L_{k}=\left\{p_{1}^{k},p_{2}^{k},\ldots ,p_{m}^{k}\right\}$, previous keyframe point cloud set $L_{k-1}=\left\{p_{1}^{k-1},p_{2}^{k-1},\ldots ,p_{m}^{k-1}\right\}$, pose transformation between two frames $T^{k-1,k}$
**output:** keyframe or not
1. for $p_{i}^{k-1}$ in $L_{k-1}$ do
2.    $N_{car}^{k-1}++$;   $N_{pole}^{k-1}++$;   $N_{building}^{k-1}++$
3. end for
4. for $p_{i}^{k}$ in $L_{k}$ do
5.    $N_{car}^{k}++$;   $N_{pole}^{k}++$;   $N_{building}^{k}++$
6. end for
7. $D_{KL}\leftarrow KL\left(N_{car}^{k-1},N_{pole}^{k-1},N_{building}^{k-1},N_{car}^{k},N_{pole}^{k},N_{building}^{k}\right)$
8. $S\leftarrow score(N_{building}^{k},N_{pole}^{k},N_{car}^{k})$
9. $w\leftarrow adaption(D_{KL},S)$
10. $angleT^{\prime },distT^{\prime }\leftarrow modify\left(angleT,distT,w\right)$
11. $x,y,z,roll,pitch,yaw\leftarrow getTransBetween(T^{k-1,k}$))
12. if(check(x,y,z,roll,pitch,yaw,$angleT^{\prime }$,$distT^{\prime }$))
13.   return false
14. end if
15. return true

## 4. Experiments and Results

To validate the advantages of our algorithm, we designed a series of experiments to evaluate and analyze the proposed method and system. The experiments are based on the KITTI dataset, and comparative experiments are conducted in three areas: associative feature filtering, keyframe selection strategy, and pose estimation results. Firstly, we tested the effectiveness of the proposed association method by comparing the number of times nearest neighbor point usage at different distances; then, we compared the number of point cloud frames successfully selected as keyframes with the total number of all point cloud frames, which showed that the method was able to increase the number of effective keyframes; lastly, we compared the performance of the SLAM system in terms of pose estimation accuracy with other SLAM systems, to verifying the advantages of our system in improving the accuracy. All experiments are executed on an experimental platform with Intel i7-8700 CPU, 32 G RAM, and Ubuntu 18.04 system environment.

### 4.1. Datasets and Evaluation Method

In order to verify the effectiveness of the proposed algorithm, we conduct experiments on the public dataset KITTI [21]. The KITTI dataset provides LiDAR data, image data, IMU/GPS data, and calibration information covering 22 sequences. The data were collected around the medium-sized city of Karlsruhe, in rural areas and on motorways, and the scenes contain objects such as vehicles, pedestrians, and buildings. We validated the experiments using 10 sequences with ground truth in the KITTI dataset. Table 1 lists information about the KITTI sequences used in the experiments. These sequences cover a variety of scenes, such as urban, rural and high-speed roads, and the length of the trajectories ranges from 394 m to 5067 m, including trajectories with and without loops. Therefore, the performance of the proposed algorithm can be fully verified.

To quantitatively evaluate the experimental results, we use Absolute Trajectory Error (ATE) as a metric to assess the accuracy of the pose estimation. ATE measures the accuracy of the system on the overall trajectory by calculating the absolute Root Mean Square Error (RMSE) between the estimated pose and the true pose for each keyframe.

### 4.2. Feature Association Comparison

To verify the performance of the feature association method, we tested the sequences 01 and 06 in KITTI dataset, respectively. In the experiments, the effectiveness of the traditional association method and the proposed method is compared by analyzing the number of times association use at different distances from the nearest neighbors.

In traditional methods, the five nearest neighbors are usually directly selected as association points to construct the residuals. Our proposed method introduces normal vector consistency to filter five points from 15 nearest neighbors as association points. To visualize the effectiveness of the method, we sort the candidate matching points according to the nearest-neighbor distance and count the number of times the serial numbers of the matching points are finally used for feature association. The formula for calculating the total number of times $N_{i}$ that a point with matching point serial number *i* is selected in the feature association process is shown below:

(23) $$N_{i}=\sum_{j=1}^{k}n_{j}$$

where $n_{j}$ denotes the number of times the point with matching point number *i* is selected in the *j*th feature association process, *k* denotes the total number of times of feature association process.

**Figure 2:**
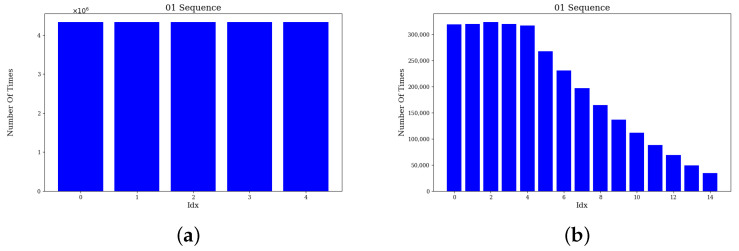
The number of times use of 01 sequence correlation points. (**a**) Traditional method. (**b**) Proposed method.

**Figure 3:**
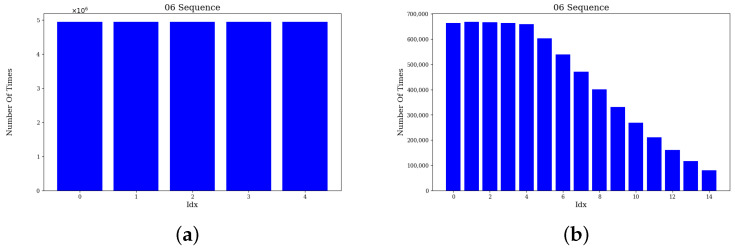
The number of times of 06 sequence correlation points. (**a**) Traditional method. (**b**) Proposed method.

Figure 2 and Figure 3 show the statistics of the number of times the match point sequence numbers in sequences 01 and 06, respectively. Figure 2a,b and Figure 3a,b indicate the statistics of the traditional and proposed methods, respectively. The horizontal axis of the figure indicates the serial number of the candidate match point and the vertical axis shows the number of times the match point is selected as a match point in the feature association phase. It can be observed that in the traditional method, only the five nearest neighbor points are selected as matching points. Whereas the proposed algorithm is not limited to the first five nearest neighbor points, the 6th to 15th nearest neighbor points are also used as matching points. Although these matching points are used less frequently than the first 5 points, they still account for a larger proportion of feature association.

To further validate the effectiveness of the method, we conducted comparative experiments on pose estimation accuracy for sequences 01 and 06. The results show that the accuracy of the proposed method is improved by 2.77% and 2.86% in these two sequences, respectively. Therefore, by introducing normal vector information, the associated points can be selected more accurately, thus improving the accuracy of feature point matching.

### 4.3. Keyframe Selection Strategy Comparison

To verify the effectiveness of the proposed keyframe selection method, we analyzed sequences 06 and 07 of the KITTI dataset. Sequence 06 has a trajectory length of 1232 m and contains a total of 1101 frames of lidar scan data. Sequence 07 has a trajectory length of 694 m and also contains 1101 frames of LiDAR scan data. The traditional method selects keyframes based on the geometric difference thresholds of translation and rotation between neighboring frames. In contrast, the proposed method evaluates the inter-frame similarity and stability by combining the semantic information to adjust the geometric thresholds for keyframe selection dynamically. Our experiments compare the traditional keyframe selection method with the proposed semantic-based improved method.

The experimental results are shown in Table 2. In sequence 06, the proposed method selects 545 keyframes, while the traditional method selects 534 keyframes, and the attitude estimation accuracy is improved by 3.64%. In sequence 07, the proposed method selects 552 keyframes, while the conventional method selects only 424 keyframes, and the pose estimation accuracy is improved by 0.38%. It can be seen that the proposed keyframe selection strategy selects significantly more keyframes than the traditional method, which indicates that the proposed approach improves the estimated pose accuracy by selecting more keyframes.

**Figure 4:**
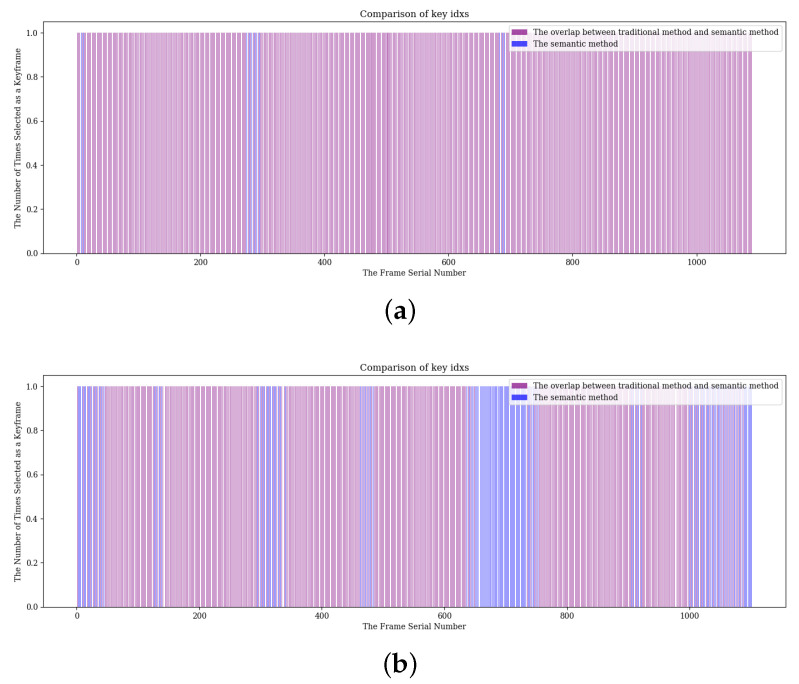
In the same sequence, the keyframe sequence numbers selected by the traditional pose change method and the method using the scene semantic information were compared. (**a**) sequence 06. (**b**) sequence 07.

To visualize the distribution of keyframes in the SLAM process more intuitively, we counted the distribution of keyframe ordinal numbers of the two methods in the trajectory. As shown in Figure 4, red identifies the traditional method and blue identifies the improved method. The X-axis indicates the frame serial number, and the Y-axis indicates the number of times the keyframes are selected.

For the proposed keyframe selection method, in Sequence 06, keyframes are selected more frequently between frames 200 to 300 and 600 to 700, where the robot is performing a turning operation and the viewpoint undergoes a continuous transformation in these regions. Similarly, Sequence 07 has significantly more frequent keyframe selection between frames 300 to 400 and in the range of 500 to 700 frames, and there are continuous turns in these regions. These observations show that the proposed method exhibits more sensitive and accurate key frame selection in regions with changing environmental characteristics, effectively improving the accuracy of system attitude estimation.

### 4.4. Pose Estimation Comparison

To assess the precision and stability of the proposed LIO-SAM++, we contrast its performance with classical SLAM, and the comparison model is as follows:

(1) LEGO-LOAM [1] filters noise through point cloud segmentation, matches lidar point cloud frames using surface and corner features, and reduces cumulative drift errors using the loop closure detection module, thus enhancing the accuracy of overall localization accuracy.
(2) LIO-SAM [2] performs pose estimation by feature matching between point cloud frames and local maps and constructs a factor graph for back-end optimization.
(3) FAST-LIO [3] gives iterative Kalman filtering to estimate the pose transform and updates the feature point map based on the state estimation results.
(4) SUMA++ [4] introduces semantic information based on the SUMA, filters the dynamic targets through the semantic difference between the observation at the current moment and the previous observation, and introduces semantic constraints into the residuals, thus generating semantic maps and improving localization accuracy in complex environments. Notably, SUMA++ also integrates RangeNet++ for semantic segmentation.

We performed a series of experiments across various sequences of the KITTI dataset to assess the localization accuracy of diverse SLAM algorithms. Table 3 summarises the absolute trajectory errors of different SLAM algorithms in eight scenes. The experimental outcomes indicate that the proposed LIO-SAM++ has an average absolute trajectory error of 6.56m over multiple sequences, which outperforms other algorithms overall. Compared to LEGO-LOAM, LIO-SAM, FAST-LIO, and SUAM++, the improvements are 13.1%, 8.6%, 15.2%, and 19.4%, indicating that the proposed algorithm has higher localization accuracy in most scenes. It is worth noting that LEGO-LOAM shows significant drift or even fails in some cases in many sequences, which is mainly due to the point cloud segmentation module affecting the number of extracted features, resulting in the inability to associate features accurately. In sequences 05 and 07, due to the presence of a large number of dynamic objects, the trajectories generated by the LIO-SAM++ drifted to some extent, mainly due to incorrectly associated feature points generated by the dynamic objects. Nevertheless, LIO-SAM++ still exhibits strong trajectory consistency overall, indicating that it still has high robustness and stability when dealing with diverse scenes.

For a more in-depth analysis, we chose sequences 02, 09 and 10 from the KITTI dataset for trajectory comparison, which cover diverse scenarios such as urban and rural areas. We show the trajectory comparisons of different algorithms and the deviation from the ground truth, respectively.

**Figure 5:**
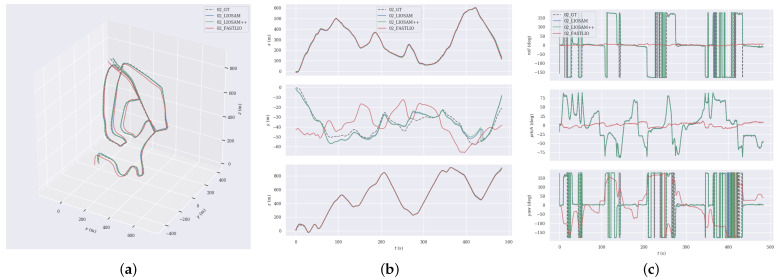
Comparative analysis of the estimated trajectories against the GT in sequence 02. (**a**) trajectories. (**b**) position. (**c**) rotation.

Figure 5 depict the comparison results of trajectories, relative position, and relative rotations for each algorithm in sequence 02.Sequence 02 is a 5067-m-long urban scene sequence containing a large number of curves. In this scene, the trajectories generated by the LIO-SAM++ algorithm are very close to the ground truth, especially in the curved sections, where it significantly outperforms other algorithms.In comparison, the trajectories of the other algorithms and the deviations of other algorithms are larger. That is because the keyframe thresholds set by LIO-SAM++ through the adaptive understanding of the environment can better adapt to the scene changes, avoiding the omission of valid keyframes when traveling in the curves and thus enabling more accurate estimation of the trajectory.

**Figure 6:**
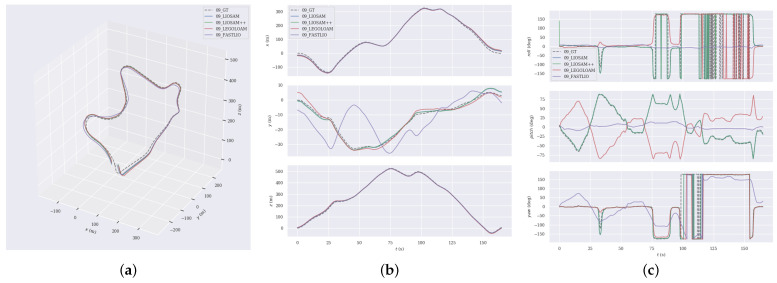
Comparative analysis of the estimated trajectories against the GT in sequence 09. (**a**) trajectories. (**b**) position. (**c**) rotation.

In Sequence 09, the vehicle navigates through a rural road environment, and there are many objects like vegetation, buildings, and roads, as well as significant elevation changes along the route. Figure 6 displays the trajectory comparison outcomes for this sequence, where the FAST-LIO has significant trajectory errors.In contrast, the trajectories obtained by LEGO-LOAM and LIO-SAM are largely coincident, and the LIO-SAM++ algorithm behaves closer to the true values.

**Figure 7:**
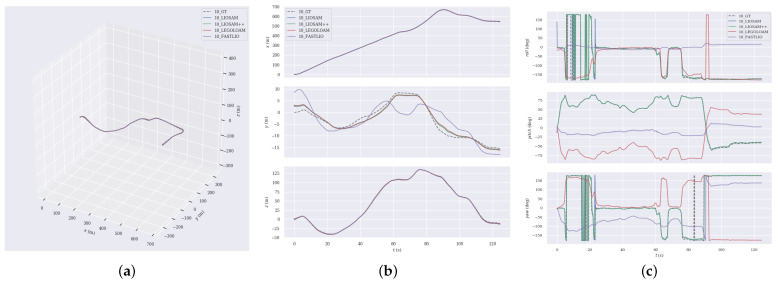
Comparative analysis of the estimated trajectories against the GT in sequence 10. (**a**) trajectories. (**b**) position. (**c**) rotation.

Sequence 10 is also in complex rural road conditions and contains a large number of dynamic objects. Figure 7 illustrates that LIO-SAM++ surpasses other methods, with its trajectory almost exactly aligning with the real trajectories, especially in the error control of translational and rotational poses. This is due to the more stable and accurate features provided by the proposed feature association method, which ensures the robustness of the pose estimation through normal vector and semantic consistency constraints, thus reducing the system drift.

In addition, from the intermediate subplots (labeled ‘b’) of Figure 5, Figure 6 and Figure 7, it can be seen that the different algorithms have more consistent trajectory estimation in the x-axis and z-axis directions. Still, there is a significant difference in the y-axis direction. This is due to the lower measurement resolution of LiDAR in the y-axis direction and less observation information from the ground, resulting in insufficient y-axis constraints. Nevertheless, the proposed LIO-SAM++ still shows strong robustness in the y-axis.

Overall, the experiment results verify the effectiveness of the proposed algorithm in various scenes and demonstrate that it can improve the accuracy of the SLAM system.

## 5. Conclusions

In this paper, we propose a lidar-inertial semantic SLAM algorithm with association optimization and keyframe selection. The algorithm obtains more accurate feature associations by geometrically modeling the local point cloud and filtering out associated points that are not in the same plane. Then, semantic consistency is added to the estimation problem using the semantic label as a constraint weighting term, and this approach takes semantic inaccuracies fully into account. In the optimization process, the distribution of semantic labels is used to calculate the inter-frame differences and the degree of stability of the current frame to focus on the changing characteristics of the scene, thus achieving a more reasonable keyframe selection.

Experimental validation on the KITTI Odometry dataset demonstrates that our algorithm enhances the precision of pose estimation and simultaneously generates clearly structured semantic point cloud maps. Even though the proposed framework provides reliable results, the work of semantic mapping still faces some challenges. Currently, the only open-source dataset with semantic labeling of LiDAR frames is SemannticKITTI, which limits the generality of the point cloud segmentation network used. In addition, the adaptability in dynamic environments has not been fully validated. In subsequent research, we aim to explore the potential of semantic information further to handle dynamic objects better and, consequently, to deal with more complex real-world situations.

## Figures and Tables

**Table 1:** Scene information of each sequence in KITTI dataset.

Sequence	Number of Scans	Trajectory Length (m)
01	1101	2453
02	4661	5067
04	271	393
05	2761	2205
06	1101	1232
07	1101	694
09	1591	1705
10	1201	919

**Table 2:** Comparison of keyframe selection methods.

Sequence	Number of Frames	Number of Key Frames (Traditional Method)	Number of Key Frames (Proposed Method)	ATE/m
06	1101	532	545	14.001
07	1101	424	552	0.437

**Table 3:** Comparison of absolute trajectory error on the KITTI datasets (RMSE of ATE [m]).

Sequence	LEGO-LOAM	LIO-SAM	FAST-LIO	SUMA++	LIO-SAM++ (Ours)
01	-	20.31	14.96	-	19.76
02	-	9.21	18.87	30.25	6.25
04	0.39	0.25	0.16	0.94	0.25
05	3.29	0.81	3.14	1.11	0.87
06	26.84	14.51	4.90	1.14	13.99
07	2.02	0.43	0.84	0.93	0.44
09	11.76	9.73	14.08	14.65	8.95
10	2.12	2.17	4.94	8.81	1.97
Average	7.55	7.18	7.74	8.14	6.56

## Data Availability

The dataset used in the paper is the public KITTI Odometry, which can be downloaded at: https://www.cvlibs.net/datasets/kitti/eval_odometry.php (accessed on 24 November 2024).
